# Applied machine learning in Alzheimer's disease research: omics, imaging, and clinical data

**DOI:** 10.1042/ETLS20210249

**Published:** 2021-12-09

**Authors:** Ziyi Li, Xiaoqian Jiang, Yizhuo Wang, Yejin Kim

**Affiliations:** 1 Department of Biostatistics, The University of Texas MD Anderson Cancer Center, Houston, TX, U.S.A.; 2 School of Biomedical Informatics, The University of Texas Health Science Center, Houston, TX, U.S.A.

**Keywords:** Alzheimer's disease, classification, deep learning, drug repurposing, machine learning, subtyping

## Abstract

Alzheimer's disease (AD) remains a devastating neurodegenerative disease with few preventive or curative treatments available. Modern technology developments of high-throughput omics platforms and imaging equipment provide unprecedented opportunities to study the etiology and progression of this disease. Meanwhile, the vast amount of data from various modalities, such as genetics, proteomics, transcriptomics, and imaging, as well as clinical features impose great challenges in data integration and analysis. Machine learning (ML) methods offer novel techniques to address high dimensional data, integrate data from different sources, model the etiological and clinical heterogeneity, and discover new biomarkers. These directions have the potential to help us better manage the disease progression and develop novel treatment strategies. This mini-review paper summarizes different ML methods that have been applied to study AD using single-platform or multi-modal data. We review the current state of ML applications for five key directions of AD research: disease classification, drug repurposing, subtyping, progression prediction, and biomarker discovery. This summary provides insights about the current research status of ML-based AD research and highlights potential directions for future research.

## Introduction

Alzheimer's disease (AD) impacted more than five million Americans in 2020, which has imposed a huge psychological and economic burden on patients, their families, and society [1]. For many years, only symptomatic treatments were available, as no drug existed to effectively stop or alter disease progression [2]. Recently, the first therapeutic drug, Aduhelm, was approved by the US Food and Drug Administration bringing new hope to those suffering from AD [3]. However, this drug was only shown to effectively reduce amyloid plaque, a protein surrogate for disease outcome [4]. Additional evidence on the actual treatment is still needed to confirm drug efficacy.

As a neurodegenerative disease, the complex nature of AD has been recognized since the very first report on the topic [5,6]. There are at least three layers of complexity to work through while seeking to fully understanding the disease. First, AD is heterogeneous, both etiologically and clinically [7,8]. Many past efforts have tried to delineate the number of AD distinct subtypes, but there is still no consensus [9]. Knowledge of potential subtyping may bring opportunities to identify subject-specific mediation and treatment approaches [10]. Second, AD is a progressive disease with a long prodromal phase [11]. Previous findings show that the disease etiology may start years or even decades before symptom onset [12]. Early diagnosis is especially desirable to manage disease progression [13]. Third, multi-faceted factors are involved in the disease. Numerous studies have recognized that no single genetic or environmental factor has enough accuracy to predict the onset of AD in a clinical setting [14,15].

Recent AD research uses novel technology or multi-modal data to understand the disease from various aspects including genomics, transcriptomics, metabolites, imaging, and clinical features [16,17]. These explorations have transformed our understanding of AD and provided new opportunities to improve our ability to manage disease progression and identify potential treatments. Meanwhile, these data are usually high in volume and data complexity, imposing challenges to data integration and analysis that traditional computation tools may not be able to fully address [18–20].

Machine learning (ML) methods have grown rapidly over recent decades and have been applied widely in the context of precision medicine [21]. The latest developments in deep learning (DL) methods further increase the ability and accuracy of analyzing large-scale complicated data [22,23]. Currently, ML methods have been explored and used in many health-related applications, as reported in cancers [24,25], cardiovascular disease [26], HIV/AIDS [27], and other health-related areas. Reviews are also available for application of ML methods using specific datatypes, for example, single cell RNA-seq [28,29], medical imaging [30–32], and multi-omic data integration [33,34]. In contrast, the application of ML methods in AD are still in their embryonic stage. Due to the complicated nature of AD, however, ML methods have the potential to further improve our understanding of the disease.

**Figure 1.**
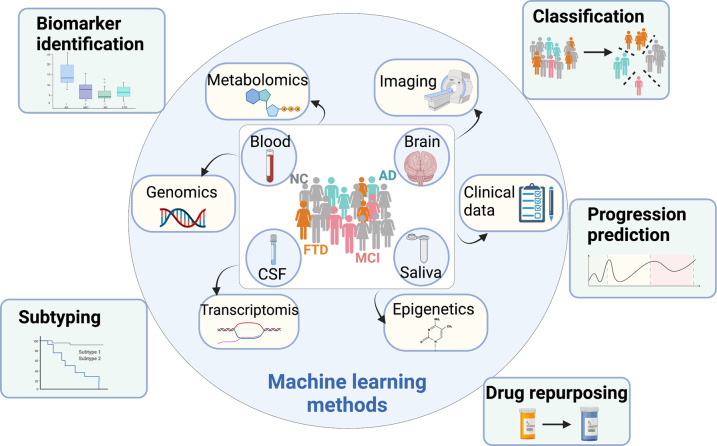
A summary of topics covered in this mini review.

This mini-review provides a focused discussion of ML applications to AD using data from one or multi-platforms. To the best of our knowledge, this is the first systematic review of ML methods in AD research covering a wide range of applications. Specifically, we consider the following five aspects of applications (summarized in Figure 1): (i) disease classification, (ii) subtyping, (iii) prediction of disease progression, (iv) biomarker discovery, and (v) drug repurposing. The papers reviewed are also summarized in Supplementary Material S1. We aim to summarize the applied ML methods for each aspect concisely and provide readers a quick head-start in the related direction.

## Backgrounds

### Machine learning

ML is one of the most common subsets of artificial intelligence concerned with how computers tackle complex learning tasks from past data [35]. As a burgeoning interdisciplinary field, ML is born at the intersection of statistics, which explores general concepts of inference, and computer science, which develops faster programming algorithms [36]. The key difference between conventional statistical methodologies and ML is that the latter draws inference and allows decisions to be made from examples rather than programming explicitly with rules [37].

Based on the nature of desired outcomes provided to the learning algorithms, ML can be supervised [38], unsupervised [39], or reinforced [40]. In supervised learning, the algorithm is presented with a labeled training set and aims to find a pattern mapping the input data to the output data. In unsupervised learning, the training set is unlabeled and only contains the input data. In other words, the algorithm does not predict output data, but rather aims to find unique structures in the input data. In reinforcement learning, the algorithm is trained in a dynamic environment and is being taught by a rewards program. It gets ‘rewarded’ for correct decisions and ‘punished’ for wrong decisions. In this way, the algorithm learns from experience instead of data.

Machines generally take more examples than humans to learn the same task, as machines lack common sense. On the other hand, machines can process a massive wealth of data. In this respect, ML algorithms advance in taking in tens of thousands of clinical data stored in electronic health records (EHRs) and hundreds of millions of genomic data, as well as imaging data generated from the laboratory experiments [36].

### Available data resources for AD research

The huge economic and psychological burden, as well as the serious damage done by AD cannot be ignored. Over decades, research communities have collected data from AD or asymptotic AD patients to investigate disease-associated factors. In Table 1, we provide a summary of available data resources for AD research. This is not an exhaustive collection, but a list of some commonly used data sources to provide some references for researchers new to the field. To facilitate diverse therapeutic target identification, various omics data platforms, including AMP-AD, M2OVE-AD, Psych-AD, and ROSMAP, provide transcriptomic and genomic variants obtained from animal models and human cohorts. Agora is a data platform where AD researchers nominate such therapeutic targets. Drug repurposing data resources, such as AD Atlas and DRIAD, provide online tool to identify repurposable FDA-approved drugs via network analysis and enrichment analysis. Potential drugs identified by genomic approach are to be tested using *in vivo* or *in vitro* models and AlzPED is the database that stores the *in vivo* efficacy of drug candidates. From clinical perspective, several consortiums, such as ADNI and NACC, have collected real-world data of cognitive normal, prodromal, and AD patients with imaging, blood-based biomarkers, and neuropsychological tests.

**Table 1:** Summary of publicly available data resources for AD research

Data type	Source
Omics	AD Knowledge Portal (e.g. AMP-AD [41], M2OVE-AD [42], Psych-AD [43], ROSMAP [44], National Institute on Aging Genetics of Alzheimer's Disease Data Storage Site (NIAGADS) of GSE5281 [45], GSE36980 [46]
Nominated target and drug repurposing	Agora [47], AD Atlas [48], DRIAD [49]
Preclinical efficacy data	AlzPED [50]
Real-world patient data (neuropsychological tests and imaging)	ADNI [18], National Alzheimer's Coordinating Center or NACC [51], OASIS [52,53], DementiaBank [54], CCC [55], TADPOLE [56]
Knowledge repository	AMP-AD [41], AlzForum [57]

## ML tasks in AD research

### Disease classification

AD patients usually have a long prodromal phase when effective treatment strategies may be applied to delay or alter the onset of the symptoms. To effectively and accurately identify AD patients or the subjects who are at high risk of developing AD, a series of studies have been conducted to classify AD from mild cognitive impairment (MCI) and from healthy controls.

Imaging data, including magnetic resonance imaging (MRI), positron emission tomography (PET) and electroencephalography (EEG), are the most commonly used data types for classification studies. Early works to classify AD patients from normal controls mainly adopt traditional ML methods, such as support vector machine [58], multi-layer perceptron [58,59], autoencoder [60], and convolutional neural network [61,62]. These methods generally can achieve a classification accuracy ∼0.9. A few modified versions of traditional methods, such as Bayesian Gaussian process logistic regression [63,64] and elastic net regularized logistic regression [65], have also demonstrated favorable performance with classification accuracies ∼0.95. A review paper by Khan and Usman [66] provides a summary of 11 papers using ML for early diagnosis of AD, including some of the papers presented here.

With the booming development of DL, researchers also have started to apply related techniques to classifying imaging data for AD diagnosis. For example, DeepAD [67] used the Inception architecture on MRI data to achieve a prediction accuracy of more than 0.98. The Inception architecture was originally built by Google, and it can learn the non-linear function by changing how convolutional layers are connected [68]. Along the same line, Hon and Khan [69] adapted two additional popular DL architectures, VEGG16 and InceptionV4, to MRI data. Additionally, they innovatively used transfer learning to greatly reduce the required training size to ∼10% of that in Szegedy et al. [68], while still achieving a comparable performance. These models are typically designed for 2D data only. Currently many studies work on extending CNNs to 3D data which is more common in neuroimaging (MRI, PET) [70].

ML methods also have been used for analyzing neuropsychological data, such as the acoustic, semantic, and syntactic elements of speech records. When extracted features are available, methods have been applied to classify AD patients from controls with an accuracy of ∼0.80; these methods include decision tree [55], support vector machine [71], and random forest [72]. When raw language text is of interest, ML methods like decision tree and bagging have been applied, but they only achieved an accuracy of ∼0.83 [73,74]. Conversely, DL models showed their advantage by obtaining more than 0.90 accuracy in the same settings. Example applications include deep-deep neural network language model [55,75] and convolutional neural network-long short-term memory model [76]. Lyu [77] provided a more detailed review for the application of ML methods in neuropsychological data from AD patients.

Another advantage of ML and DL methods is the ability to integrate data from multiple platforms for disease classification. Previous works that used multi-modal data include combining different platforms of imaging data, e.g. MRI and PET by stacked autoencoder [63,78], MRI and FDG-PET by deep neural network [79]; combining imaging data and patient features, e.g. MRI and cerebrospinal fluid markers by support vector machine [80], MRI and clinical features by local weighted learning [81] and by XGBoost [81,82], MRI and neuropsychological data as well as biomarkers by multi-task deep neural network [83]; and combining data from multiple omics platforms, e.g. gene expression and DNA methylation data by deep neural network [84].

A review of the above classification studies reveals all of the above classification studies reveals that integrating multiple data sources does not necessarily provide higher accuracy than using a single data platform. Studies using imaging data, however, do tend to have better prediction performance than those without imaging data. Likewise, DL models tend to produce higher accuracy. Also of note, classifying MCI patients from controls or AD from MCI are harder problems than classifying AD from controls, which always has lower accuracy (0.76 ∼ 0.87).

### Drug repurposing

Current computational AD drug repurposing has been studied from various perspectives: transcriptome, network pharmacology, and treatment effects in real-world patient observation [85].

#### Drug-induced gene expression

The transcriptomic-based strategy to drug repurposing compares drug-induced gene expression with AD gene expression [86–88]. This approach captures integrated molecular changes in AD pathology. Such methods focus on the genetic signature of drugs and disease to investigate the association between drug-induced perturbation and the disease [89–91]. Williams et al. [87] first applied the genetic signature approach to discover drugs that oppose disease-associated genes in neuronal cells. Rodriguez et al. [92] extended the work to the disease's genetic signatures from various disease stages (e.g. Braak stage) and calculated the association between a gene expression on drug-induced perturbation of neuronal cells and molecular changes in the brains of AD patients at different stages.

#### Network pharmacology

The network-based approach represents drugs’ multi-target capacity in a human interaction network and aims to estimate proximity between disease modules and drugs [93–97]. This tactic can facilitate drug repurposing by helping to identify targets and drug–target interaction prediction.

For target identification, several efforts integrate multi-omics data (e.g. metabolites, proteins, epigenetic modification, and GWAS catalog) by integrating multiple biological interactions [14,98,99]. Another line of studies uses a broader set of data associated with drugs (e.g. side effect, pharmacological hierarchy) and leverages knowledge graph representation to identify AD-related genes [100,101]. In particular, several platforms curate the multi-modal and comprehensive interactions from public data collections and experimental data generated by multiple consortiums [60,101,102].

Prior studies on drug–target interaction prediction have aimed to identify hidden interactions among drugs and proteins (i.e. putative disease target). By identifying hidden drug–target interaction, it is possible to identify existing drugs that may have new indications for AD. Hidden drug–target interaction can be revealed by finding new drug–target binding (off-target) or by integrating multi-modal interactions (on-target) [103]. The off-target approach uses biochemical properties (structural, ligand-based molecular docking) or biophysical properties (3D conformation) to predict drug–target binding [85,104]. The on-target approach uses protein–protein interactions or drug–drug similarity to estimate network proximity between entities. Predicting drug–target interactions can be facilitated by a large biological knowledgebase with multi-modal interactions (e.g. drug–target, target–target, drug–disease, drug–side effect, drug–drug, gene–functional ontology, drug–functional ontology) and use graph ML (e.g. network proximity, graph neural representation) [93,101,105–107].

#### Population-based treatment effect

This approach, based on real-world patient data, leverages large-scale patient datasets to obtain off-the-label drug efficacy via counterfactual inference [108,109]. Real-world patient data includes administrative data (EHRs, insurance claim data), clinical observational data, and clinical trials data. Several statistics and ML methodologies are applied to the patient data, such as potential treatment outcome models (or target trial) [110], and meta-analysis [108]. The techniques from causal inference, such as propensity score matching, have also been used in potential outcomes or target trial approaches [109]. The merit of the population-based approach is that it captures different drug responses in heterogeneous populations.

### Subtyping of AD

The purpose of AD/ADRD patient subtyping is to use computational approach to mine big healthcare data to identify clinically homogeneous group of patients based on their characteristics and biological markers, considering existing biomedical knowledge and clues derived from data. AD is complicated by different etiologies and a large variability of patient characteristics. Depending on comorbidity, genotype, race, and gender, different patients exhibit different degradation pathways. There is no one-size-fits-all solution to model the complexity of the patient population. AD subtyping with ML is a current research topic, and various researchers have applied different methods to gain a better understanding of the complex and heterogeneous patient population.

Alexander et al. [111] used UK primary care EHRs from the CALIBER resource to identify and characterize clinically meaningful clusters of patients using unsupervised learning approaches of multiple correspondence analysis (MCA) and K-means. Vizcarra et al. [112] validated previously published ML algorithms using convolutional neural networks (CNNs) and to determine if pathological heterogeneity may alter algorithm-derived measures using 40 cases from the Goizueta Emory Alzheimer's Disease Center brain bank, which displays an array of pathological diagnoses (including AD with and without Lewy body disease (LBD) and/or TDP-43-positive inclusions) and evaluated their levels of Aβ pathologies. Shehzad et al. [113] used individualized symptom profiles from the pooled data (clinical data from 717 people from three sources: (1) a memory clinic, (2) long-term care, and (3) an open-label trial of donepezil in vascular and mixed dementia) to train various ML models to predict dementia severity (MCI, mild dementia, moderate dementia, or severe dementia). Tsao et al. [114] combined a predictive multi-task ML method (cFSGL) with a novel ML-based multivariate morphometric surface map of the hippocampus (mTBM) to predict future cognitive scores (Alzheimer's Disease Assessment Scale cognitive scores 6, 12, 24, 36, and 48 months from baseline) of patients. Giang, Nguyen, and Tran [115] proposed a fast-multiple kernel learning framework, referred to as fMKL-DR, to optimize equations to calculate matrix chain multiplication and reduce dimensions in data space to stratify AD patients into different phases. Mar et al. [116] validated random forest models by using them to identify depressive and psychotic clusters according to their presence in the EHRs of all patients diagnosed with dementia.

Various AD subtyping studies have used different data modalities, including imaging data, clinical records, clinical notes, cognitive scores, and genetic profiles, to offer partial evidence of patient stratification, but none of these have provided deterministic characterization or a biomarker that allows researchers to separate patient populations (e.g. into fast and slow progressors). Such challenges have prevented the development of targeted clinical trials and hamper personalized health care.

### Prediction of disease progression

Predicting disease progression has two unique tasks. The first is to identify MCI or normal patients who are at higher risk of converting to AD. The second is to predict longitudinal AD-related scores. The first task is similar to the classification problem discussed in Section 2.1. However, the methods in that section usually took advantage of longitudinal observations and had a special focus on disease progression. Some studies only used baseline information to predict the MCI-to-AD conversion in the future and the ML methods they adopted include support vector machine with linear kernel [117], multi-task neural network classifier [83], logistic regression [118] and multi-kernel learning [119]. When longitudinal measurements, such as lab results and cognitive tests, were available, the problem of predicting disease progression was more complicated. A few recent works addressed this problem using advanced DL methods, including conditional restricted Boltzmann machine [120], recurrent neural network [121], and ensemble model based on stacked convolutional neural network and bidirectional long short-term memory network [122].

For the second task, a pioneering work in 2016 applied nonlinear supervised sparse regression-based random forest on the MRI data from the ADNI to predict a variety of longitudinal AD clinical scores [123]. Another recent work incorporated the multi-modal data from MRI, PET, and FDG-PET with support vector machines and predicted rates of decline in patients’ global cognition and memory [124].

Due to the complexity of the longitudinal data and the problem, most of the methods reviewed in this section were developed using multi-source data. For example, combining imaging information (MRI, PET) and cerebrospinal fluid markers [117,118,125]. Clinical biomarkers (e.g. lab results, neuropsychological) have also been analyzed together with imaging data [83]. Although statistical methods are not the focus of the current review, researchers also have adopted more complicated statistical modeling to accommodate the longitudinal observations and predict patients’ progression [126].

### Biomarker discovery

Identifying novel biomarkers to distinguish AD from MCI or normal controls is highly associated with classification and progression prediction. In fact, the first step of establishing a classification or prediction model with a large dataset is usually to select a set of informative biomarkers. For example, Challis et al. [64] selected features with the largest absolute Kendall tau correlation coefficients versus the class label. When constructing the Gaussian process logistic regression, they also applied automatic relevance determination parameterizations to down weight the contribution of less relevant features. In the study using multi-source datasets, it is even more important to perform feature selection so that a parsimonious model can be established. An example of such a study is Zhang and Shen [127]. The authors used a multi-task feature selection that selects the common subset of relevant features from each modality. Then the joint set of selected features were pooled together for later steps of classification analysis. Similarly, Park and Park [84] applied differential analysis on gene expression and DNA methylation datasets, respectively, and selected the top differentially expressed signals as the features. This feature selection procedure facilitates the establishment of deep neural network models to classify AD and normal controls. However, linking these selected features to ‘true’ biomarkers that can be used for clinical utility is still a hard problem and needs further exploration.

Motivated by these limitations, some studies also aimed to identify optimal combinations of existing biomarkers for disease progression [128–130]. Most of these methods used straight-forward models such as linear regressions or logistic regressions. Some other methods used more complicated tools. For example, Szalkai et al. [131] used association rule mining and Karaglani et al. [132] used an automatic ML pipeline of SVMs to identify optimal combinations of biomarkers. Some of these biomarker identification methods have been reviewed by Chang et al. [133]. Badhwar et al. [14] also provided reviews on the biomarker-related methods to identify imaging, metabolomics and genomics biomarkers. Those studies mainly used straight-forward methods such as logistic regression and SVM to evaluate different biomarker candidates [134,135].

## Challenges and opportunities in AD research using ML

### Heterogeneity

Individuals diagnosed with AD usually demonstrate a high level of heterogeneity in clinical trajectory, symptoms, as well as neurodegenerative biomarkers. Such heterogeneity is highly associated with the etiology of the disease, and thus it is important to take such heterogeneity into consideration for each analytical task. Some existing works have already recognized this and focused on a sub-group of AD patients with more homogenized clinical features [136–140]. The existing datasets, such as ADNI and ROSMAP, provided data for relatively large AD sample groups. However, when focusing on subgroups with specific clinical characteristics, the sample size diminishes. Compared with distinguishing AD from controls, the tasks of comparing MCI versus control or MCI versus AD are more difficult and need more samples to properly train ML methods. Moreover, the current data from different sources tend to have incompatible formats and various qualities. There is a need to collect more data to address these questions, especially more high-quality and integrated data.

In addition to increasing study sample size, another way to address heterogeneity is to use multi-omics or multi-modal data. Different sources of biomarkers (e.g. imaging features, clinical measurements, omics) provide information from different aspects. Many existing studies have already incorporated data from multiple imaging platforms or imaging data with clinical features [82,124]. However, limited studies have used multi-omics data or combined multi-omics with imaging and clinical features [14]. The power of DL in handling high-volume and multi-source data has been demonstrated in many other scenarios [141,142]. With the accumulation of data and the increasing number of available biomarker sources, incorporating multi-source data with DL models to further improve classification and predictive ability could be a promising future direction.

### Early diagnosis and identification of alterable factors

As a progressive disease with a long prodromal phase, early diagnosis and subtyping can guide clinical decision making and improve prognostic outcomes. Limited by the available resources, the majority of existing studies focus on studying AD versus controls, while only a few study control versus MCI or MCI versus AD. Admittedly, the latter two are harder problems, but still there is significant room to improve the accuracy of the existing models for distinguishing MCI versus controls or MCI versus AD for increased clinical utility [127].

In addition, identifying alterable factors is an important task that may substantially influence AD treatment and prognosis. While existing studies demonstrate that ML models using imaging data generally have higher diagnosis accuracy than using other data sources, the omics data types (e.g. genomics, metabolomics, as well epigenetics) actually provide a better chance to identify alterable gene/pathway markers or metabolite targets [84,143]. This could be another promising direction for ML and DL to play an important role in future research.

### Integrating multi-source data

Researchers integrate different sources of data when targeting different outcomes. For example, the prediction and classification studies integrate data from multiple imaging platforms [63,78] or integrate imaging data with clinical or omics data [81,82]. The drug repurposing studies more often use data from multiple omics modalities to depict the relevant biological processes [14,98,99]. Assembling the multi-source data expands the view from a single platform and may generate a more comprehensive understanding of the research objective.

However, many challenges exist for such data integration. First, combining data from different sources requires unifying the data format and controlling the data quality. To address these needs, a series of data harmonization guidelines and tools have been developed [144–146]. Most of these tools are developed in recent years and still need further validation to understand their performance. Second, more analytical methods are needed to provide rigorous data integration and analysis. A general data integration method usually requires extensive feature selection from each platform to reduce the computational burden in the data integrating step [80,127]. Usually, the feature selection is performed in an *ad hoc* way and may become a hurdle for having reproducible findings. The high volume of data also poses challenges for data storage and data sharing. Some recent reviews in this area provided more in-depth discussions [14,20].

Last but not least, incorporating sparse or incomplete datasets is another challenge often present in addressing multi-modal data analysis. For example, how to train machine learning modeling using data with missing or censored values? How to integrate high-dimensional imaging or omic data that were not collected from all of the participants. Although some efforts have been made to provide a solution [147,148], more methods are still needed to have a consensus answer across the research communities.

### Accurate identification of AD from real-world patient data

Several subtyping or drug repurposing studies have utilized administrative real-world patient data such as EHRs or insurance claim data. In spite of the EHRs’ advantages of extensively covering a population's long medical history in breadth, EHRs are mainly collected for billing, not for scholarly studies, and therefore diagnosis billing codes in EHRs are sometimes incomplete and lack details, which consequently make accurate AD identification difficult compared with observational studies with neuropsychological or imaging-based diagnosis. Differential diagnosis with related dementia (e.g. vascular dementia, Lewy body dementia, frontotemporal dementia) is also practically challenging due to heterogeneity of AD. Identifying computable AD phenotypes based on multi-modal information (e.g. co-occurrence with medication or imaging procedure, clinical notes) could help better detection of AD.

### Reproducibility of the results

Almost all of the existing studies applied the proposed methods to only one or two datasets. It is quite difficult to compare performance (e.g. accuracy, sensitivity, specificity) from studies using different datasets. Moreover, the methods tailored for existing datasets may not perform well in real clinical settings or heterogeneous populations [149]. Therefore, there is a need to systematically benchmark existing methods or future proposals and to collect more datasets that contain diverse populations from real clinical settings.

Lastly, more than half of the methodology works we reviewed in this paper did not provide their software in publicly available repositories. We believe promoting software availability is crucial in reproducible research. At the same time, a user-friendly software allows more researchers and clinicians to apply the methods in practice. 

**Figure 2.**
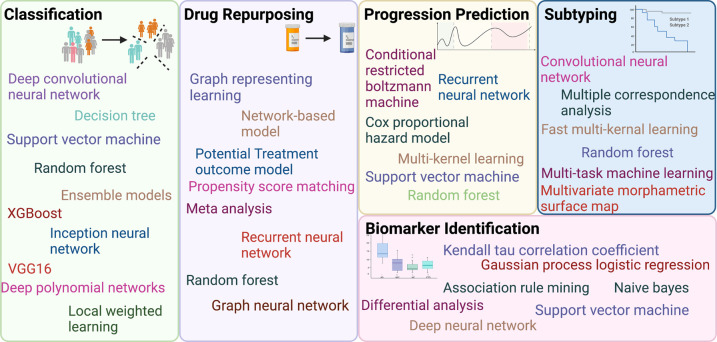
Machine learning methods applied to Alzheimer's disease research.

## Conclusion

This review provides a concise summary of ML methods applied in AD research. Figure 2 summarizes the ML methods used for different AD research areas. Written for an interdisciplinary audience, the goal of the paper is to provide up-to-date information with recent advances, useful overviews and emerging trends for the readers. Over the past decade, the field quickly embraces the power of ML for complex data analysis and integration. There is also an increasing trend using deep learning techniques to mine the high-volume and high-complicated data in AD research.

## Summary

- Massive amounts of data from different platforms for AD research pose great challenges for data analysis.
- A wide variety of ML and DL methods have been applied to classify and subtype patients, predict progression, identify biomarkers, and explore drug repurposing.
- Further efforts are needed to expand the current datasets, incorporate heterogeneity into the analysis, develop methods for addressing issues in multi-source data integration, and apply the findings in real clinical practice.

## Abbreviations

AD: Alzheimer's disease
CNNs: convolutional neural networks
DL: deep learning
EHRs: electronic health records
MCI: mild cognitive impairment
ML: machine learning
MRI: magnetic resonance imaging
PET: positron emission tomography
