# Supplementary material for: Applied machine learning in Alzheimer's disease research: omics, imaging, and clinical data
Source: Emerg Top Life Sci. 2021 Dec 9;5(6):765–77. doi: 10.1042/ETLS20210249 (PMC8786302; doi:10.1042/ETLS20210249)
Supplement: Supplementary Information [file ETLS-5-765-s1.pdf]

Name	Journal	Authors	Year	Ref	ML models
**Disease classification**					
Alzheimer’s disease Classification from Brain MRI based on transfer learning from CNN	BMEiCON-2018	Khagi et al.	2018	(1)	PCA+TSNE+KNN, NB
Deep ensemble learning for Alzheimer's disease Classification	Journal of Biomedical informatics	An et al.	2020	(2)	Ensemble methods
Classification of Alzheimer’s Disease and Parkinson’s Disease by Using Machine Learning and Neural Network Methods	2010 Second International Conference on Machine Learning and Computing	Joshi et al.	2010	(3)	Decision tree, bagging, BF tree, RF, RBF, MLP, NN
Combining MRI and CSF measures for classification of Alzheimer's disease and prediction of mild cognitive impairment conversion	NeuroImage	Westman et al.	2012	(4)	Apriori-AR mining + SVM
Multimodal EEG, MRI and PET data fusion for Alzheimer's disease diagnosis	EMBC 2010 conference	Polikar et al.	2010	(5)	Ensemble based decision fusion
Deep learning-based pipeline to recognize Alzheimer's disease using fMRI data	Future Technologies Conference	Sarraf et al.	2016	(6)	Deep convolutional neural network
Gaussian process classification of Alzheimer's disease and mild cognitive impairment from resting-state fMRI	NeuroImage	Challis et al.	2015	(7)	Bayeisan Gaussian process Logistic regression
Classification of mild cognitive impairment and Alzheimer’s Disease with machine-learning techniques using 1H Magnetic Resonance Spectroscopy data	Expert system with Applications	Munteanu et al.	2015	(8)	MLP
Diagnosis and monitoring of Alzheimer's patients using classical and deep learning techniques	Expert system with Applications	Raza et al.	2019	(9)	DNN, SVM

Using high-dimensional machine learning methods to estimate an anatomical risk factor for Alzheimer's disease across imaging databases	NeuroImage	Casanova et al.	2018	(10)	Elastic net regularized logistic regression
Towards Alzheimer's Disease Classification through Transfer Learning	2017 IEEE International confererence on Bioinformatics and biomedicine	Hon and Khan	2017	(11)	Inception V4 Transfer learning, VGG16
Predicting alzheimer’s disease: a neuroimaging study with 3d convolutional neural networks,	arXiv	Payan and Montana	2015	(12)	3DConv
Natural image bases to represent neuroimaging data	International conference on machine learning	Gupta et al.	2013	(13)	Sparse autoencoder+convolutional NN
Machine learning-based method for personalized and cost-effective detection of Alzheimer's disease	IEEE TRANSACTIONS ON BIOMEDICAL ENGINEERING	Escudero et al.	2013	(14)	Local weighted learning
Multimodal and multiscale deep neural networks for the early diagnosis of Alzheimer's disease using structural MR and FDG-PET images	Scientific report	Lu et al.	2018	(15)	Multimodel and multiscale deep neural network
Deepad: Alzheimer s disease classification via deep convolutional neural networks using mri and fmri	bioRxiv	Sarraf et al.	2016	(16)	DeepAD: Inception
Multimodal neuroimaging feature learning with multimodal stacked deep polynomial networks for diagnosis of Alzheimer's disease	IEEE J. Biomed. Health Inf.	Shi et al.	2018	(17)	Multimodel stacked deep polynomial netowrks
Early diagnosis of alzheimer's disease with deep learning	2014 IEEE 11th international symposium on	Liu et al	2014	(18)	stacked AE and a softmax output layer

	biomedical imaging (ISBI)				
Developing a Machine Learning Workflow to Explain Black-box Models for Alzheimer's Disease Classification	HEALTHINF	Bloch and Friedrich	2021	(19)	XGBoost, RF, CART
A parameter-efficient deep learning approach to predict conversion from mild cognitive impairment to Alzheimer's disease	Neuroimage	Spasov et al.	2019	(20)	Deep-learning-based
Multi-modal multi-task learning for joint prediction of multiple regression and classification variables in Alzheimer's disease	NeuroImage	Zhang and Shen	2012	(21)	Multi-task feature selection plus multi-modal support vector machine
Multimodal classification of Alzheimer's disease and mild cognitive impairment	NeuroImage	Zhang et al.	2011	(22)	SVM
Prediction of Alzheimer's disease based on deep neural network by integrating gene expression and DNA methylation dataset	Expert system with Applications	Park et al.	2020	(23)	(23)DNN
Linguistic features identify Alzheimer’s disease in narrative speech	Journal of Alzheimer's Disease	Fraser et al.	2016	(24)	Machine learning
A new machine learning method for identifying Alzheimer's disease	Simulation Modeling Practice and Theory	Liu et al.	2020	(25)	Logistic regression; Decision tree; Bagging; MLP
Features and machine learning classification of connected speech samples from patients with autopsy proven Alzheimer's disease with and without additional vascular pathology	Journal of Alzheimer's Disease	Rentoumi et al.	2014	(26)	Machine learning

Progression prediction					
Machine learning for comprehensive forecasting of Alzheimer's disease progression	Scientific reports	Fisher et al.	2019	(27)	Conditional Restricted Boltzmann Machine
Predictive markers for AD in a multi-modality framework: an analysis of MCI progression in the ADNI population	Neuroimage	Hinrichs et al.	2011	(28)	Multi-kernel learning
Predicting sporadic Alzheimer’s disease progression via inherited Alzheimer’s disease-informed machine-learning	Alzheimer's & Dementia: Journal Alzheimer's Assoc	Franzmeier et al.	2019	(29)	SVM predict rates of decline in global cognition and memory
Biomarker-based prediction of progression in MCI: comparison of AD signature and hippocampal volume with spinal fluid amyloid-beta and tau	Front Aging Neurosci	Dickerson et al.	2013	(30)	logistic regression model
Brain beta-amyloid measures and magnetic resonance imaging atrophy both predict time-to-progression from mild cognitive impairment to Alzheimer’s disease	Brain	Jack et al.	2010	(31)	Cox proportional hazards
Incremental value of biomarker combinations to predict progression of mild cognitive impairement to Alzheimer's dementia	Alzheimers Res Ther	Frolich et al.	2017	(32)	support vector machine with linear kernel
Predicting Alzheimer's disease progression using multi-modal deep learning approach	Scientific reports	Lee et al.	2019	(33)	RNN
Multimodal multitask deep learning model for Alzheimer’s disease progression detection based on time series data	Neurocomoputing	El-Sappagh et al.	2020	(34)	Emsemble model based on stacked CNN and Bidirectional long short-term memory network

Modeling disease progression via multisource multitask learners: a case study with alzheimer’s disease	IEEE Transactions on Neural Networks and Learning Systems	Nie et al.	2016	(35)	Multisource multi-task learning
Modeling disease progression via multi-task learning	NeuroImage	Zhou et al.	2013	(36)	Multi-task learning based on temporal group Lasso regularizer
Longitudinal clinical score prediction in alzheimer’s disease with soft-split sparse regression based random forest	Neurobiology of aging	Huang et al.	2016	(37)	nonlinear supervised sparse regression–based random forest
A parameter-efficient deep learning approach to predict conversion from mild cognitive impairment to alzheimer’s disease	Neuroimage	Spasov et al.	2019	(20)	multi-task neural network classifier
Prognosis prediction model for conversion from mild cognitive impairment to Alzheimer's disease created by integrative analysis of multi-omics data	Alzheimer's research	Shigemizu et al.	2020	(38)	cox proportional hazards
**Biomarker identification**					
Identifying combinatorial biomarkers by association rule mining in the CAMD Alzheimer’s database	ArchGerontologyGeriatr	Szalkai et al.	2017	(39)	association rule mining
Accurate Blood-Based Diagnostic Biosignatures for Alzheimer’s Disease via Automated Machine Learning	Journal of Clinical Medicine	Karaglani et al.	2020	(40)	SVM
Machine Learning and Novel Biomarkers for the Diagnosis of Alzheimer’s Disease	International Journal of Molecular Sciences	Chang et al.	2021	(41)	support vector machine, logistic regression, random forest, and naïve bayes

NMR analysis of the human saliva metabolome distinguishes dementia patients from matched controls	Molecular BioSystems	Figueira et al.	2016	(42)	multivariate regression
Metabolomics analyses of saliva detect novel biomarkers of Alzheimer's disease	Journal of Alzheimer's Disease	Huan et al.	2018	(43)	Area under ROC curve
Multi-modal multi-task learning for joint prediction of multiple regression and classification variables in Alzheimer's disease.	Neuroimage	Zhang and Shen	2012	(21)	Multi-modal multi-task learning
Plasma metabolite profiles of Alzheimer's disease and mild cognitive impairment	Journal of Porteome Research	Wang et al.	2014	(44)	ROC curve and logistic regression
What success can teach us about failure: the plasma metabolome of older adults with superior memory and lessons for Alzheimer's disease	Neurobiol Aging	Mapstone et al.	2017	(45)	ROC curve and logistic regression
Blood-based metabolic signatures in Alzheimer's disease	Alzheimers Dementia	de Leeuw et al.	2017	(46)	Penalized regression and network models
**Subtyping**					
Using unsupervised learning to identify clinical subtypes of Alzheimer's disease in electronic health records	IOS Press: Digital Personalized Health and Medicine	Alexander et al.	2020	(47)	MCA and K-means
Validation of machine learning models to detect amyloid pathologies across institutions	Acta Neuropathologica Communications	Vizcarra et al.	2020	(48)	CNN
Use of patient-reported symptoms from an online symptom tracking tool for dementia severity staging: Development and validation of a machine learning approach	Journal of Medical Internet Research	Shehzad et al.	2020	(49)	Support vector machine, k-nearest neighbor, random forest, neural network, logistic regression, stochastic gradient boosting

Feature selective temporal prediction of Alzheimer's disease progression using hippocampus surface morphometry	Brain and Behavior	Tsao et al.	2017	(50)	Multi-task machine learning framework
Stratifying patients using fast multiple kernel learning framework: case studies of Alzheimer's disease and cancers	BMC Medical Informatics and Decision Making	Giang et al.	2020	(51)	Fast-multiple kernel learning framework
Estimation of the epidemiology of dementia and associated neuropsychiatric symptoms by applying machine learning to real-world data	Psychiatry and Mental Health	Mar et al.	2021	(52)	Random forest
**Drug repurposing**					
Machine learning identifies candidates for drug repurposing in Alzheimer's disease	Nature Communications	Rodriguez et al.	2021	(53)	Logistic regression, support vector machines, boosted random forest models, and two-layer fully connected neural networks
Harnessing endophenotypes and network medicine for Alzheimer's drug repurposing	Medical Research Reviews	Fang et al.	2020	(54)	Network-based approach
A deep learning framework for high-throughput mechanism-driven phenotype compound screening	Nature Machine Intelligence	Pham et al.	2020	(55)	Mechanism-driven neural network-based method
DrugComboRanker: drug combination discovery based on target network analysis	BMC Bioinformatics	Huang et al.	2014	(56)	Network analysis
Driver network as a biomarker: systematic integration and network modeling of multi-omics data to derive driver signaling pathways for drug combination prediction	BMC Bioinformatics	Huang et al.	2019	(57)	An integrated pipeline of algorithms, including bootstrap aggregating-based Markov random field, weighted co-expression network analysis and supervised regulatory network learning

Context-sensitive network analysis identifies food metabolites associated with Alzheimer's disease: an exploratory study	BMC Medical Genomics	Chen et al.	2019	(58)	Network analysis
Drug Repositioning for Alzheimer's Disease Based on Systematic 'omics' Data Mining	PLOS One	Zhang et al.	2016	(59)	Data mining
Alzheimer's disease in the omics era (Review)	Clinical Biochemistry	Sancesario et al.	2018	(60)	Review
Novel drug target identification for the treatment of dementia using multi-relational association mining	Nature Scientific reports	Nguyen et al.	2015	(60, 61)	Association mining
AlzGPS: a genome-wide positioning systems platform to catalyze multi-omics for Alzheimer's drug discovery	BMC Alzheimer's Research & Therapy	Zhou et al.	2021	(62)	Bioinformatics method
HENA, heterogeneous network-based data set for Alzheimer's disease. Scientific Data	Nature Scientific Data	Sügis et al.	2019	(63)	Graph convolutional networks
Utilizing graph machine learning within drug discovery and development	Briefings in Bioinformatics	Gaudelet et al.	2021	(64)	Graph machine learning (GML)
In silico drug repositioning for the treatment of Alzheimer's disease using molecular docking and gene expression data	RSC Advances	Xie et al.	2016	(65)	bioinformatics method
deepDR: a network-based deep learning approach to in silico drug repositioning	BMC Bioinformatics	Zeng et al.	2019	(66)	A network-based deep learning approach
DrugGenEx-Net: a novel computational platform for systems pharmacology and gene expression-based drug repurposing	BMC Bioinformatics	Issa et al.	2016	(67)	bioinformatics method
Drug repurposing with network reinforcement	BMC Bioinformatics	Nam et al.	2019	(68)	A network-based reinforment algorithm

Evidence for benefit of statins to modify cognitive decline and risk in Alzheimer's disease	Alzheimer's Research & Therapy	Geifman et al.	2017	(69)	meta analysis
Sex and Race Differences in the Association Between Statin Use and the Incidence of Alzheimer Disease	JAMA Neurology	Zissimopoulos et al.	2017	(70)	Epidemiology method
Using Big Data to Emulate a Target Trial When a Randomized Trial Is Not Available	American Journal of Epidemiology	Hernán et al.	2016	(71)	Causal inference
Insights into Computational Drug Repurposing for Neurodegenerative Disease (Review)	Trends in Pharmacological Sciences	Paranjpe et al.	2019	(72)	Review
Bioinformatics methods in drug repurposing for Alzheimer's disease	Briefings in Bioinformatics	Siavelis et al.	2016	(73)	Bioinformatics method
Drug repurposing for Alzheimer's disease based on transcriptional profiling of human iPSC-derived cortical neurons	Translational Psychiatry	Williams et al.	2019	(68)	Bioinformatics method
Discovery and preclinical validation of drug indications using compendia of public gene expression data	Sci Transl Med	Sirota et al.	2011	(74)	Bioinformatics method
The Connectivity Map: Using Gene-Expression Signatures to Connect Small Molecules	Genes, and Disease. Science	Lamb J.	2006	(75)	Bioinformatics method
L1000CDS: LINCS L1000 characteristic direction signatures search engine	NPJ Syst Biol Appl	Duan et al.	2016	(76)	Bioinformatics method
L1000FWD: fireworks visualization of drug induced transcriptomic signatures	Bioinformatics	Wang et al.	2018	(77)	Bioinformatics method

1. Khagi B, Lee CG, Kwon G-R, editors. Alzheimer's disease Classification from Brain MRI based on transfer learning from CNN. 2018 11th biomedical engineering international conference (BMEiCON); 2018: IEEE.

2. An N, Ding H, Yang J, Au R, Ang TF. Deep ensemble learning for Alzheimer's disease classification. Journal of biomedical informatics. 2020;105:103411.
3. Joshi S, Shenoy D, Simha GV, Rrashmi P, Venugopal K, Patnaik L, editors. Classification of Alzheimer's disease and Parkinson's disease by using machine learning and neural network methods. 2010 Second International Conference on Machine Learning and Computing; 2010: IEEE.
4. Westman E, Muehlboeck JS, Simmons A. Combining MRI and CSF measures for classification of Alzheimer's disease and prediction of mild cognitive impairment conversion. Neuroimage. 2012;62(1):229-38.
5. Polikar R, Tilley C, Hillis B, Clark CM, editors. Multimodal EEG, MRI and PET data fusion for Alzheimer's disease diagnosis. 2010 Annual International Conference of the IEEE Engineering in Medicine and Biology; 2010: IEEE.
6. Sarraf S, Tofighi G. Deep Learning-based Pipeline to Recognize Alzheimerʹs Disease using fMRI Data.
7. Challis E, Hurley P, Serra L, Bozzali M, Oliver S, Cercignani M. Gaussian process classification of Alzheimer's disease and mild cognitive impairment from resting-state fMRI. NeuroImage. 2015;112:232-43.
8. Munteanu CR, Fernandez-Lozano C, Mato Abad V, Pita Fernández S, Álvarez-Linera J, Hernández-Tamames JA, et al. Classification of mild cognitive impairment and Alzheimer’s Disease with machine-learning techniques using 1H Magnetic Resonance Spectroscopy data. Expert Syst Appl. 2015;42(15):6205-14.
9. Raza M, Awais M, Ellahi W, Aslam N, Nguyen HX, Le-Minh H. Diagnosis and monitoring of Alzheimer's patients using classical and deep learning techniques. Expert Systems with Applications. 2019;136:353-64.
10. Casanova R, Barnard RT, Gaussoin SA, Saldana S, Hayden KM, Manson JE, et al. Using high-dimensional machine learning methods to estimate an anatomical risk factor for Alzheimer's disease across imaging databases. Neuroimage. 2018;183:401-11.
11. Hon M, Khan NM. Towards Alzheimer's disease classification through transfer learning. 2017 IEEE International Conference on Bioinformatics and Biomedicine (BIBM). 2017.
12. Payan A, Montana G. Predicting Alzheimer's disease: a neuroimaging study with 3D convolutional neural networks. arXiv [csCV]. 2015.
13. Gupta A, Ayhan M, Maida A. Natural Image Bases to Represent Neuroimaging Data. In: Dasgupta S, McAllester D, editors. Proceedings of the 30th International Conference on Machine Learning; 2013. Atlanta, Georgia, USA: PMLR; 2013. p. 987-94.
14. Escudero J, Ifeachor E, Zajicek JP, Green C, Shearer J, Pearson S, et al. Machine learning-based method for personalized and cost-effective detection of Alzheimer's disease. IEEE Trans Biomed Eng. 2013;60(1):164-8.
15. Lu D, Popuri K, Ding GW, Balachandar R, Beg MF, Alzheimer’s Disease Neuroimaging I. Multimodal and Multiscale Deep Neural Networks for the Early Diagnosis of Alzheimer's Disease using structural MR and FDG-PET images. Sci Rep. 2018;8(1):5697.
16. Disease Neuroimaging Initiative As. DeepAD: Alzheimer's disease classification via deep convolutional neural networks using MRI and fMRI. BioRxiv. 2016.

17. Shi J, Zheng X, Li Y, Zhang Q, Ying S. Multimodal Neuroimaging Feature Learning With Multimodal Stacked Deep Polynomial Networks for Diagnosis of Alzheimer's Disease. IEEE J Biomed Health Inform. 2018;22(1):173-83.
18. Khan A, Usman M. Early Diagnosis of Alzheimer's Disease using Machine Learning Techniques - A Review Paper. Proceedings of the 7th International Joint Conference on Knowledge Discovery, Knowledge Engineering and Knowledge Management. 2015.
19. Bloch L, Friedrich C. Developing a Machine Learning Workflow to Explain Black-box Models for Alzheimer’s Disease Classification. Proceedings of the 14th International Joint Conference on Biomedical Engineering Systems and Technologies. 2021.
20. Spasov S, Passamonti L, Duggento A, Liò P, Toschi N. A parameter-efficient deep learning approach to predict conversion from mild cognitive impairment to Alzheimer’s disease.
21. Zhang D, Shen D, Alzheimer's Disease Neuroimaging I. Multi-modal multi-task learning for joint prediction of multiple regression and classification variables in Alzheimer's disease. Neuroimage. 2012;59(2):895-907.
22. Zhang D, Wang Y, Zhou L, Yuan H, Shen D, Alzheimer's Disease Neuroimaging I. Multimodal classification of Alzheimer's disease and mild cognitive impairment. Neuroimage. 2011;55(3):856-67.
23. Park C, Ha J, Park S. Prediction of Alzheimer's disease based on deep neural network by integrating gene expression and DNA methylation dataset. Expert Syst Appl. 2020;140:112873.
24. Fraser KC, Meltzer JA, Rudzicz F. Linguistic features identify Alzheimer’s disease in narrative speech. Journal of Alzheimer's Disease. 2016;49(2):407-22.
25. Liu L, Zhao S, Chen H, Wang A. A new machine learning method for identifying Alzheimer's disease. Simulation Modelling Practice and Theory. 2020;99:102023.
26. Rentoumi V, Raoufian L, Ahmed S, de Jager CA, Garrard P. Features and machine learning classification of connected speech samples from patients with autopsy proven Alzheimer's disease with and without additional vascular pathology. J Alzheimers Dis. 2014;42 Suppl 3:S3-17.
27. Fisher CK, Smith AM, Walsh JR, Coalition Against Major D, Abbott AfARAsAAsFoAAPLPB-MSCCPICFIEL, Company FH-LRLFRIGIGJ, et al. Machine learning for comprehensive forecasting of Alzheimer's Disease progression. Sci Rep. 2019;9(1):13622.
28. Hinrichs C, Singh V, Xu G, Johnson SC, Alzheimers Disease Neuroimaging I. Predictive markers for AD in a multi-modality framework: an analysis of MCI progression in the ADNI population. Neuroimage. 2011;55(2):574-89.
29. Franzmeier N, Koutsouleris N, Benzinger T, Goate A, Karch CM, Fagan AM, et al. Predicting sporadic Alzheimer's disease progression via inherited Alzheimer's disease-informed machine-learning. Alzheimers Dement. 2020;16(3):501-11.
30. Dickerson B. Biomarker-based prediction of progression in MCI: comparison of AD signature and hippocampal volume with spinal fluid amyloid-β and tau. Frontiers in Aging Neuroscience. 2013;5.

31. Jack Jr CR, Wiste HJ, Vemuri P, Weigand SD, Senjem ML, Zeng G, et al. Brain beta-amyloid measures and magnetic resonance imaging atrophy both predict time-to-progression from mild cognitive impairment to Alzheimer’s disease. Brain. 2010;133(11):3336-48.
32. Frölich L, Peters O, Lewczuk P, Gruber O, Teipel SJ, Gertz HJ, et al. Incremental value of biomarker combinations to predict progression of mild cognitive impairment to Alzheimer's dementia. Alzheimers Res Ther. 2017;9(1):84.
33. Lee G, Nho K, Kang B, Sohn K-A, Kim D, for Alzheimer’s Disease Neuroimaging I. Predicting Alzheimer's disease progression using multi-modal deep learning approach. Sci Rep. 2019;9(1):1952.
34. El-Sappagh S, Abuhmed T, Riazul Islam SM, Kwak KS. Multimodal multitask deep learning model for Alzheimer’s disease progression detection based on time series data. Neurocomputing. 2020;412:197-215.
35. Nie L, Zhang L, Meng L, Song X, Chang X, Li X. Modeling Disease Progression via Multisource Multitask Learners: A Case Study With Alzheimer’s Disease. IEEE Transactions on Neural Networks and Learning Systems. 2017;28(7):1508-19.
36. Zhou J, Liu J, Narayan VA, Ye J, Initiative AsDN. Modeling disease progression via multi-task learning. NeuroImage. 2013;78:233-48.
37. Huang L, Jin Y, Gao Y, Thung K-H, Shen D, Alzheimer's Disease Neuroimaging I. Longitudinal clinical score prediction in Alzheimer's disease with soft-split sparse regression based random forest. Neurobiol Aging. 2016;46:180-91.
38. Shigemizu D, Akiyama S, Higaki S, Sugimoto T, Sakurai T, Boroevich KA, et al. Prognosis prediction model for conversion from mild cognitive impairment to Alzheimer’s disease created by integrative analysis of multi-omics data. Alzheimer's research & therapy. 2020;12(1):1-12.
39. Szalkai B, Grolmusz VK, Grolmusz VI, Coalition Against Major D. Identifying combinatorial biomarkers by association rule mining in the CAMD Alzheimer's database. Arch Gerontol Geriatr. 2017;73:300-7.
40. Karaglani M, Gourlia K, Tsamardinos I, Chatzaki E. Accurate Blood-Based Diagnostic Biosignatures for Alzheimer's Disease via Automated Machine Learning. J Clin Med Res. 2020;9(9).
41. Chang C-H, Lin C-H, Lane H-Y. Machine Learning and Novel Biomarkers for the Diagnosis of Alzheimer’s Disease. International Journal of Molecular Sciences. 2021;22(5):2761.
42. Figueira J, Jonsson P, Adolfsson AN, Adolfsson R, Nyberg L, Öhman A. NMR analysis of the human saliva metabolome distinguishes dementia patients from matched controls. Molecular BioSystems. 2016;12(8):2562-71.
43. Huan T, Tran T, Zheng J, Sapkota S, MacDonald SW, Camicioli R, et al. Metabolomics analyses of saliva detect novel biomarkers of Alzheimer’s disease. Journal of Alzheimer's Disease. 2018;65(4):1401-16.
44. Wang G, Zhou Y, Huang F-J, Tang H-D, Xu X-H, Liu J-J, et al. Plasma metabolite profiles of Alzheimer's disease and mild cognitive impairment. J Proteome Res. 2014;13(5):2649-58.

45. Mapstone M, Lin F, Nalls MA, Cheema AK, Singleton AB, Fiandaca MS, et al. What success can teach us about failure: the plasma metabolome of older adults with superior memory and lessons for Alzheimer's disease. Neurobiol Aging. 2017;51:148-55.
46. de Leeuw FA, Peeters CFW, Kester MI, Harms AC, Struys EA, Hankemeier T, et al. Blood-based metabolic signatures in Alzheimer's disease. Alzheimers Dement. 2017;8:196-207.
47. Alexander N, Alexander DC, Barkhof F, Denaxas S. Using unsupervised learning to identify clinical subtypes of Alzheimer's disease in electronic health records. Studies in health technology and informatics. 2020;270:499-503.
48. Vizcarra JC, Gearing M, Keiser MJ, Glass JD, Dugger BN, Gutman DA. Validation of machine learning models to detect amyloid pathologies across institutions. Acta Neuropathol Commun. 2020;8(1):59.
49. Shehzad A, Rockwood K, Stanley J, Dunn T, Howlett SE. Use of patient-reported symptoms from an online symptom tracking tool for dementia severity staging: Development and validation of a machine learning approach. J Med Internet Res. 2020;22(11):e20840.
50. Tsao S, Gajawelli N, Zhou J, Shi J, Ye J, Wang Y, et al. Feature selective temporal prediction of Alzheimer's disease progression using hippocampus surface morphometry. Brain Behav. 2017;7(7):e00733.
51. Giang T-T, Nguyen T-P, Tran D-H. Stratifying patients using fast multiple kernel learning framework: case studies of Alzheimer’s disease and cancers. BMC Medical Informatics and Decision Making. 2020;20(1).
52. Mar J, Gorostiza A, Arrospide A, Larrañaga I, Alberdi A, Cernuda C, et al. Estimation of the epidemiology of dementia and associated neuropsychiatric symptoms by applying machine learning to real-world data. Rev Psiquiatr Salud Ment (Engl Ed). 2021.
53. Rodriguez S, Hug C, Todorov P, Moret N, Boswell SA, Evans K, et al. Machine learning identifies candidates for drug repurposing in Alzheimer’s disease. Nat Commun. 2021;12(1):1-13.
54. Fang J, Pieper AA, Nussinov R, Lee G, Bekris L, Leverenz JB, et al. Harnessing endophenotypes and network medicine for Alzheimer's drug repurposing. Med Res Rev. 2020.
55. Pham T-H, Qiu Y, Zeng J, Xie L, Zhang P. A deep learning framework for high-throughput mechanism-driven phenotype compound screening. bioRxiv. 2020.
56. Huang L, Li F, Sheng J, Xia X, Ma J, Zhan M, et al. DrugComboRanker: drug combination discovery based on target network analysis. Bioinformatics. 2014;30(12):i228-36.
57. Huang L, Brunell D, Stephan C, Mancuso J, Yu X, He B, et al. Driver network as a biomarker: systematic integration and network modeling of multi-omics data to derive driver signaling pathways for drug combination prediction. Bioinformatics. 2019;35(19):3709-17.
58. Chen Y, Xu R. Context-sensitive network analysis identifies food metabolites associated with Alzheimer’s disease: an exploratory study. BMC medical genomics. 2019;12(1):133-42.

59. Zhang M, Schmitt-Ulms G, Sato C, Xi Z, Zhang Y, Zhou Y, et al. Drug Repositioning for Alzheimer's Disease Based on Systematic 'omics' Data Mining. PLoS One. 2016;11(12):e0168812.
60. Sancesario GM, Bernardini S. Alzheimer's disease in the omics era. Clin Biochem. 2018;59:9-16.
61. Nguyen T-P, Priami C, Caberlotto L. Novel drug target identification for the treatment of dementia using multi-relational association mining. Sci Rep. 2015;5(1):1-13.
62. Zhou Y, Fang J, Bekris LM, Kim YH, Pieper AA, Leverenz JB, et al. AlzGPS: a genome-wide positioning systems platform to catalyze multi-omics for Alzheimer's drug discovery. Alzheimers Res Ther. 2021;13(1):1-13.
63. Sügis E, Dauvillier J, Leontjeva A, Adler P, Hindie V, Moncion T, et al. HENA, heterogeneous network-based data set for Alzheimer's disease. Scientific Data. 2019;6(1):1-18.
64. Gaudelet T, Day B, Jamasb AR, Soman J, Regep C, Liu G, et al. Utilizing graph machine learning within drug discovery and development. Brief Bioinform. 2021.
65. Xie H, Wen H, Qin M, Xia J, Zhang D, Liu L, et al. In silico drug repositioning for the treatment of Alzheimer's disease using molecular docking and gene expression data. RSC Advances. 2016;6(100):98080-90.
66. Zeng X, Zhu S, Liu X, Zhou Y, Nussinov R, Cheng F. deepDR: a network-based deep learning approach to in silico drug repositioning. Bioinformatics. 2019;35(24):5191-8.
67. Issa NT, Kruger J, Wathieu H, Raja R, Byers SW, Dakshanamurthy S. DrugGenEx-Net: a novel computational platform for systems pharmacology and gene expression-based drug repurposing. BMC Bioinformatics. 2016;17(1):202.
68. Williams G, Gatt A, Clarke E, Corcoran J, Doherty P, Chambers D, et al. Drug repurposing for Alzheimer's disease based on transcriptional profiling of human iPSC-derived cortical neurons. Transl Psychiatry. 2019;9(1):220.
69. Geifman N, Brinton RD, Kennedy RE, Schneider LS, Butte AJ. Evidence for benefit of statins to modify cognitive decline and risk in Alzheimer's disease. Alzheimer's Research & Therapy. 2017;9(1).
70. Zissimopoulos JM, Barthold D, Brinton RD, Joyce G. Sex and Race Differences in the Association Between Statin Use and the Incidence of Alzheimer Disease. JAMA Neurol. 2017;74(2):225-32.
71. Hernán MA, Robins JM. Using Big Data to Emulate a Target Trial When a Randomized Trial Is Not Available. Am J Epidemiol. 2016;183(8):758-64.
72. Paranjpe MD, Taubes A, Sirota M. Insights into Computational Drug Repurposing for Neurodegenerative Disease. Trends Pharmacol Sci. 2019;40(8):565-76.
73. Siavelis JC, Bourdakou MM, Athanasiadis EI, Spyrou GM, Nikita KS. Bioinformatics methods in drug repurposing for Alzheimer's disease. Brief Bioinform. 2016;17(2).
74. Sirota M, Dudley JT, Kim J, Chiang AP, Morgan AA, Sweet-Cordero A, et al. Discovery and preclinical validation of drug indications using compendia of public gene expression data. Sci Transl Med. 2011;3(96):96ra77.

75. Lamb J. The Connectivity Map: Using Gene-Expression Signatures to Connect Small Molecules, Genes, and Disease. Science. 2006;313(5795):1929-35.
76. Duan Q, Reid SP, Clark NR, Wang Z, Fernandez NF, Rouillard AD, et al. L1000CDS: LINCS L1000 characteristic direction signatures search engine. NPJ Syst Biol Appl. 2016;2.
77. Wang Z, Lachmann A, Keenan AB, Ma'ayan A. L1000FWD: fireworks visualization of drug-induced transcriptomic signatures. Bioinformatics. 2018;34(12):2150-2.
